# Circadian clock-dependent increase in salivary IgA secretion modulated by sympathetic receptor activation in mice

**DOI:** 10.1038/s41598-017-09438-0

**Published:** 2017-08-18

**Authors:** Misaki Wada, Kanami Orihara, Mayo Kamagata, Koki Hama, Hiroyuki Sasaki, Atsushi Haraguchi, Hiroki Miyakawa, Atsuhito Nakao, Shigenobu Shibata

**Affiliations:** 1 Laboratory of Physiology and Pharmacology, School of Advanced Science and Engineering, Waseda University, Shinjuku-ku, Tokyo 162-8480 Japan; 2 Waseda Institute for Advanced Study, Waseda University, 1-6-1 Nishi-Waseda, Shinjuku-ku, Tokyo 169-8050 Japan; 3 Department of Immunology, Faculty of Medicine, University of Yamanashi, Yamanashi, Japan

## Abstract

The salivary gland is rhythmically controlled by sympathetic nerve activation from the suprachiasmatic nucleus (SCN), which functions as the main oscillator of circadian rhythms. In humans, salivary IgA concentrations reflect circadian rhythmicity, which peak during sleep. However, the mechanisms controlling this rhythmicity are not well understood. Therefore, we examined whether the timing of parasympathetic (pilocarpine) or sympathetic (norepinephrine; NE) activation affects IgA secretion in the saliva. The concentrations of saliva IgA modulated by pilocarpine activation or by a combination of pilocarpine and NE activation were the highest in the middle of the light period, independent of saliva flow rate. The circadian rhythm of IgA secretion was weakened by an SCN lesion and *Clock* gene mutation, suggesting the importance of the SCN and *Clock* gene on this rhythm. Adrenoceptor antagonists blocked both NE- and pilocarpine-induced basal secretion of IgA. Dimeric IgA binds to the polymeric immunoglobulin receptor (pIgR) on the basolateral surface of epithelial cells and forms the IgA-pIgR complex. The circadian rhythm of *Pigr* abundance peaked during the light period, suggesting pIgR expression upon rhythmic secretion of IgA. We speculate that activation of sympathetic nerves during sleep may protect from bacterial access to the epithelial surface through enhanced secretion of IgA.

## Introduction

Mammals possess circadian clock systems that control various physiological phenomena such as body temperature, sleep-wake cycles, and liver metabolism^1, 2^. Circadian clock systems are organized by a central clock called the suprachiasmatic nuclei (SCN)^3^, and by peripheral clocks located in many peripheral organs^4, 5^. In addition to this system, biological functions of metabolism and the immune system are also known to affect circadian rhythms^6, 7^.

IgA is a type of antibody that works mainly in the mucosal immune system. It is abundant in the mucus of saliva and the small intestine^8^. Since plasma cells produce IgA in the salivary glands, there is a large amount of IgA in saliva. Therefore, IgA plays an important role as the first line of defense in oral immunity^9^. Monomers of IgA form dimeric IgA (dIgA) through the J chain. This dIgA binds the polymeric immunoglobulin receptor (pIgR) on the basolateral surface of epithelial cells and forms the IgA-pIgR complex. The IgA-pIgR complex is transported to the lumen from the basolateral surface. Proteolytic cleavage occurs at the apical surface, and a fragment of pIgR becomes a secretory component (SC) that binds dIgA. In this way, secretory IgA (sIgA) combines with other SCs, and free SCs are released. As a result, sIgA binds to luminal bacteria and prevents them from accessing the epithelial surface^9^. Therefore, a reduction in salivary IgA levels allows bacterial access to the epithelial surface and leads to various diseases such as upper respiratory tract infections (URTI) and periodontal disease^10^. A few studies demonstrated that salivary IgA concentrations display diurnal variations in human experiments, and concentrations peak during sleep^11, 12^. However, the underlying mechanism of this diurnal variation is unknown. Therefore, signaling processes modulating IgA secretion may be controlled by circadian rhythms.

Because it is difficult to obtain an adequate amount of saliva from mice under normal conditions, some experiments used pilocarpine for parasympathetic stimulation and norepinephrine for sympathetic stimulation^13^. Saliva secretion is known to decrease following an adrenoceptor agonist injection compared to that upon injections with pilocarpine^14^. Previous studies have demonstrated that the submandibular gland expresses clock genes, which show robust circadian rhythms^15, 16^. Rhythmical *Clock* gene expression in the salivary gland is controlled by sympathetic activation via the SCN^17^. In addition, both mRNA and protein expression of adrenoceptors in the submandibular glands were reported to show circadian rhythm^18, 19^. Therefore, the timing of administration of adrenoceptor agonist injections may affect the secretion of IgA in saliva. In addition, we examined whether the SCN clock directly controls time-dependent IgA secretion via adrenoceptor activation or is indirectly controlled by the adrenal gland through sympathetic regulation. We aimed to investigate how sympathetic nerve activation affects salivary IgA secretion rhythms through control of the biological clock.

## Results

### Salivary IgA secretion increases during the light phase

**Figure 1:**
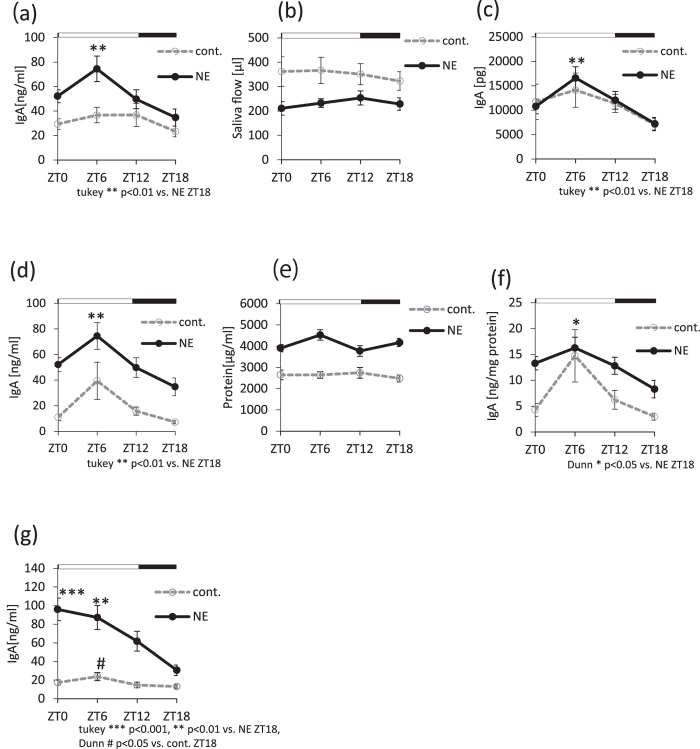
The circadian rhythm dynamics of salivary IgA secretion. (**a**) Salivary IgA secretion rhythms in the case of administration of either pilocarpine (control) or a mixture of pilocarpine and norepinephrine (NE) (n = 8–10). (**b**) Saliva flow rhythms in control versus NE groups (n = 8–10). (**c**) Salivary IgA volume rhythms. Data were calculated by multiplying the results from Fig. 1a and b (n = 8–10). (**d**) Salivary IgA concentration rhythms in control versus NE groups (control, n = 4; NE, n = 9–10). (**e**) Total protein concentration rhythms in saliva (control, n = 4; NE, n = 9–10). (**f**) Salivary IgA concentration rhythms were normalized to total protein concentration (control, n = 4; NE, n = 9–10). (**g**) Salivary IgA concentration rhythms in mice fasted for 24 hours (n = 9–12). Values are shown as the means ± SEM. (**a,c,d**) **p < 0.01, NE group ZT6 vs. ZT18 (one-way ANOVA with Tukey post-hoc test). (**f**) *p < 0.05, NE group ZT6 vs. ZT18 (Kruskal-Wallis test with Dunn post-hoc test). (**g**) ***p < 0.001 **p < 0.01, NE group ZT0, ZT6 vs. ZT18 (one-way ANOVA with Tukey post-hoc test), ^#^p < 0.05, cont. group ZT6 vs. ZT18 (Kruskal-Wallis test with Dunn post-hoc test).

We investigated whether salivary IgA secretion reflects circadian rhythms. Submandibular glands are regulated by both the sympathetic and parasympathetic nervous systems^14^. Therefore, we used pilocarpine to stimulate the parasympathetic nerves and NE to stimulate the sympathetic nerves. We observed a significant increase in IgA concentration during the light phase in the NE group, but not in the control group, as assessed by one-way ANOVA and Kruskal-Wallis test (Fig. 1a,d, Supplemental Table S1). Cosinor analysis revealed significant but weak rhythmicity in control groups, whereas NE groups showed strong rhythmicity (supplemental Table S2). The mice used in Fig. 1a–c are different from those in Fig. 1d–f, since we performed independent experiments to confirm the findings.

The saliva flow demonstrated weak circadian rhythm with peak at ZT4.8 (Zeitgeber time, ZT0 represents the start of the light period and ZT12 represents the start of the dark period) for the control and ZT11.4 for NE groups (Supplemental Table S2, Fig. 1b). The total amount of salivary IgA displayed a similar rhythm (Supplemental Table S2, Fig. 1c) as that observed for IgA concentrations (Fig. 1a). Because the stimulation of the sympathetic nerve triggers saliva protein secretion^14^, we next investigated whether total protein concentrations in saliva show a circadian rhythm. Protein concentrations did not display a circadian rhythm (Supplemental Table S2, Fig. 1e). Therefore, salivary IgA concentrations were normalized by the total protein concentration in the sample. However, this still showed a clear oscillating rhythm (supplemental Table S2, Fig. 1f) that was similar to that for IgA concentrations alone (Fig. 1d). We showed representative data for the diurnal variations of the protein concentrations, which set ratio 1 at ZT0 in saliva (Supplemental Fig. S1a). A portion of the data in Fig. 1a and d are also used in Supplemental Fig. S1b and a, respectively. In both the control and NE groups, the protein concentration ratio did not demonstrate any significant circadian rhythm (Supplemental Fig. S1a, Supplemental Table S2). However, the diurnal variation of the salivary IgA ratio increased during the light phase compared to the protein concentration ratio (Supplemental Fig. S1b, Supplemental Table S2).

To investigate whether feeding rhythm, chewing action, or amylase secretion affect salivary IgA secretion, we measured salivary IgA concentrations in mice after a 24-hour fasting period. IgA concentrations showed similar rhythms as those observed in non-fasting mice (Fig. 1g). However, the rhythm peaks in both the control and NE groups appeared 2–3 hours in advance (Fig. 1g, Supplemental Table S2) compared to the those in the free feeding group (Fig. 1a,d). These results suggest that salivary IgA secretion displays circadian rhythm. Moreover, this rhythm is not affected by saliva flow, total protein in saliva, feeding rhythm, or chewing action. Therefore, we next assessed the IgA concentration in the submandibular gland, and found that it showed weak circadian rhythm with peak at ZT3.6 (Supplemental Fig. S2, Supplemental Table S2). These results suggest that the rhythm of salivary IgA secretion is partially related to IgA concentrations in the submandibular gland.

### The rhythm of salivary IgA secretion is lost after SCN lesion

**Figure 2:**
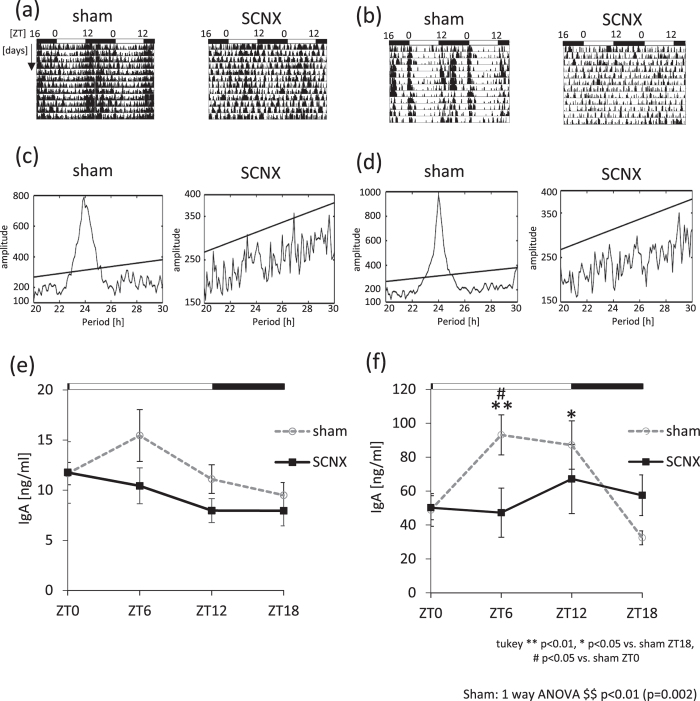
The circadian rhythms of salivary IgA secretion in SCN-lesioned mice. (**a**,**b**) Representative double-plotted actograms of locomotor activity for one sham mouse and one SCNX mouse after pilocarpine (**a**) or NE administration (**b**). (**c,d**) Representative Chi-squared periodogram analysis of behavioral data for one sham mouse and one SCNX mouse after pilocarpine (**c**) or NE (**d**) administration. Straight lines in each figure indicate statistical significance. (**e**,**f**) Salivary IgA concentration rhythms in non-SCN-lesioned (sham) and SCN-lesioned (SCNX) after pilocarpine (**e**) or NE administration (**f**) (n = 4–6). Values are shown as mean ± SEM. (**f**) ^$$^p < 0.01, sham group (one-way ANOVA). **p < 0.01, *p < 0.05 vs. sham ZT18. ^#^p < 0.05 vs. sham ZT0.

Because salivary IgA secretion shows a circadian rhythm, we examined whether salivary IgA secretion is controlled by the SCN. To investigate the influence of the central circadian clock on salivary IgA concentrations, we lesioned the SCN (SCNX) and compared IgA concentrations with those in the sham-operated group. We confirmed the success of the SCNX surgery using actograms for both the pilocarpine (Fig. 2a,c) and NE (Fig. 2b,d) groups. A representative histological analysis of the SCN lesion is shown in Supplemental Fig. S3, demonstrating that the SCN was completely destroyed. We prepared two sets of SCN-lesion groups (pilocarpine and NE) to account for the possibility of recovery after the SCN lesion. We found that the SCNX group still had rhythmicity of salivary IgA secretion in both the pilocarpine (Fig. 2e) and NE (Fig. 2f) groups. However, the rhythmicity and amplitude, as evaluated using the cosinor method, were dampened in the SCN lesioned group compared to that in the sham group (Supplemental Table S2). These results suggest that the rhythm of salivary IgA secretion is highly controlled by SCN.

### Salivary IgA concentrations are increased by sympathetic nerve agonists and decreased by sympathetic nerve antagonists

**Figure 3:**
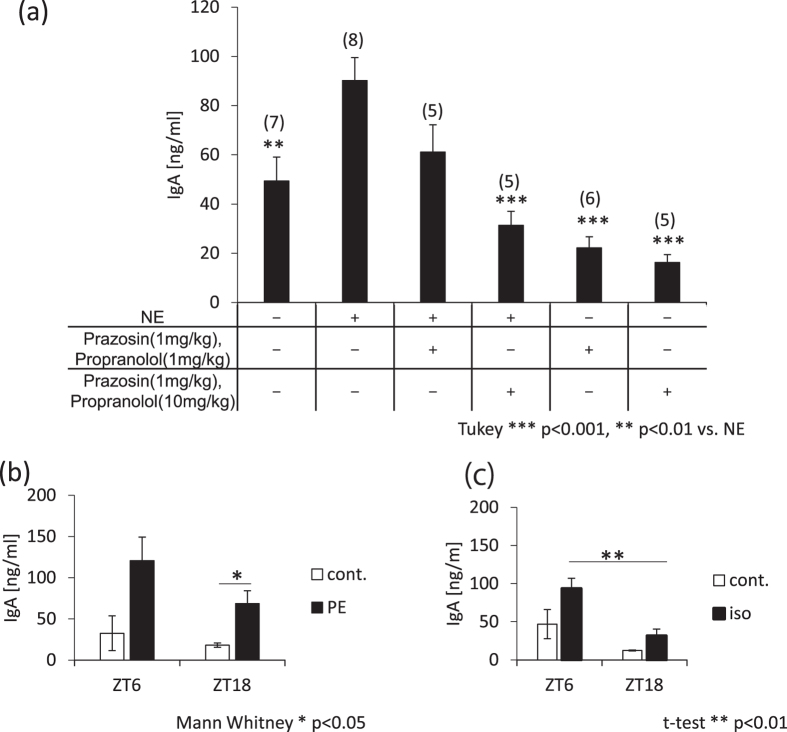
Effects of sympathetic nerve agonists/antagonists on salivary IgA secretion. (**a**) Salivary IgA concentrations after application of the sympathetic nervous system agonist (norepinephrine 1 mg/kg) and/or antagonist (prazosin 1 mg/kg, propranolol 1 mg/kg, or 10 mg/kg) with pilocarpine. “+” indicates the reagent that was mixed and administered to mice, “−” indicates the reagent that was not administered to mice. (**b**,**c**) Salivary IgA concentrations when the α receptor (phenylephrine, 5 mg/kg; the numbers in parentheses are the number of samples) (**b**) or β receptor (isoproterenol, 5 mg/kg; n = 4–5) (**c**) were stimulated at ZT6 or ZT18 (n = 3–5). Values were shown as means ± SEM. (**a**) ***p < 0.001, **p < 0.01, vs. the NE(+)-Prazosin(−)-Propranolol(−) group (one-way ANOVA with Tukey post-hoc test). (**b**) *p < 0.05, ZT18 group cont. vs. PE (Mann-Whitney test). (**c**) **p < 0.01, iso group ZT6 vs. ZT18 (Student’s t-test).

It has been suggested that the submandibular gland responds to sympathetic nervous stimulation^17^ (Fig. 1a,d). Therefore, we investigated the relationship between the sympathetic nervous system and the regulation of salivary IgA concentrations. We administered agonists and antagonists of NE at ZT6, since the salivary IgA concentration peaks at this time point (Fig. 1a). The salivary IgA concentration increased significantly with agonist treatment, and decreased with antagonist treatment (Fig. 3a). Antagonists significantly blocked the NE-induced increase in salivary IgA concentrations. In addition, both α receptor agonist phenylephrine and β receptor agonist isoproterenol increased salivary IgA concentrations at ZT6 and ZT18. However, the observation that the salivary IgA concentration is highest at the light phase was also observed in both agonists-administered groups (Fig. 3b,c). Both phenylephrine and isoproterenol showed a circadian pattern of IgA secretion (Supplemental Fig. S4a,b, Supplemental Table S2) similar to NE-induced secretion. These results suggest that the sympathetic nerve activation-induced increase of salivary IgA secretion is caused by an activation of α, β, or both receptors.

### Sensitivity of the salivary glands to sympathetic nerve stimulation is higher during the light phase

**Figure 4:**
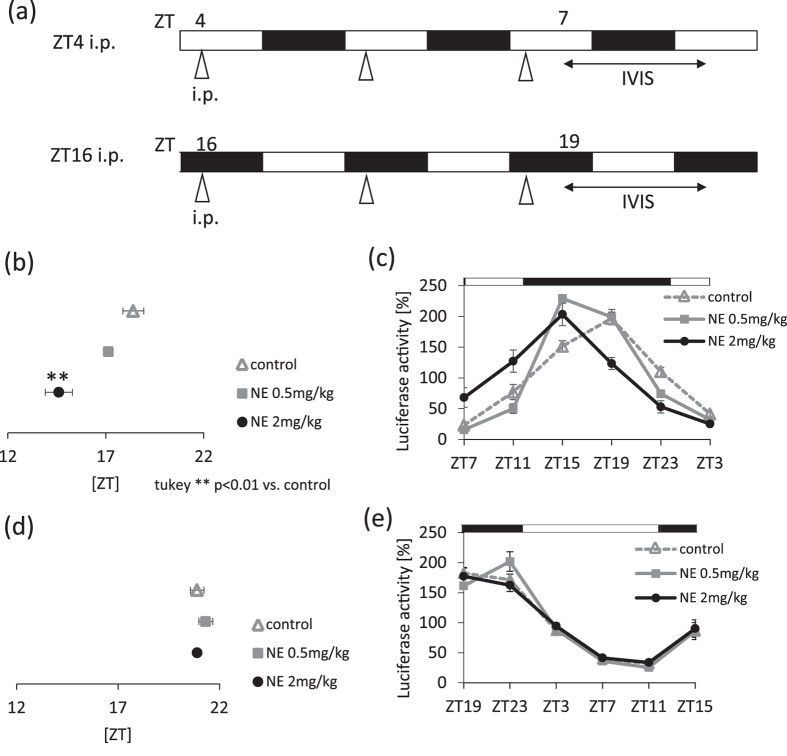
Sensitivity of the submandibular gland clock to sympathetic nerve stimulation, as evaluated by IVIS in PER2::LUC mice. (**a**) Experimental schedule. White and black bars indicate 12 hours of light and dark. Triangles under the white and black bars indicate the time at which the reagents were administered intraperitoneally. Bi-directional arrows under the white and black bars indicate the period of measurement using IVIS. (**b**,**c**) Average peaks of phase of PER2::LUC rhythms (**b**) and PER2::LUC expression rhythms (**c**) in submandibular glands after mice were administered the reagent at ZT4 for 3 days (n = 3–6). (**d**,**e**) Average phase peaks of PER2::LUC rhythms (**d**) and PER2::LUC expression rhythms (**e**) in submandibular glands after mice were administered the reagent at ZT16 for 3 days (n = 4–8). Values were shown as the means ± SEM. (**b**) **p < 0.01, control vs. NE 2 mg/kg (one-way ANOVA with Tukey post-hoc test).

*Clock* genes, which are reportedly expressed in the submandibular gland, demonstrate circadian rhythms^15, 20^. We show that salivary IgA secretion rhythms appear to be controlled by sympathetic activation through the SCN. Sympathetic stimulation causes circadian fluctuations and resulted in a circadian change in salivary IgA secretion. To investigate the submandibular gland’s sensitivity to and the time frame for sympathetic stimulation, we administrated NE during the light phase (ZT4) or the dark phase (ZT16), and compared the phase shift for *Per2* gene abundance in the submandibular gland. When NE was administrated at ZT4, the *Per2* phase advanced in a concentration-dependent manner (Fig. 4b,c, Supplemental Table S2). However, when NE was administrated at ZT16, the *Per2* phase was unaffected (Fig. 4d,e). In all groups, the circadian rhythmicity of *Per2* abundance, as evaluated by cosinor analysis, was maintained in vehicle- or NE- treated mice (Supplemental Table S2).

Next, we examined whether NE directly affected the submandibular gland function. Under *in vitro* conditions, NE administration at the rising phase of *Per2* rhythm significantly advanced the *Per2* rhythm phase without affecting the amplitude (Supplemental Fig. S5a–c). NE administration at ZT4 *in vitro* caused an increase in *Per1* gene abundance but not abundance of the *Per2* gene in the submandibular gland (Supplemental Fig. S5d,e). These results suggest that the submandibular gland has a higher sensitivity to sympathetic stimulation through direct action during the light phase than during the dark phase. In other words, there is a time window during which the submandibular gland responds to external NE stimulation. Our results suggest that ZT4 is the approximate time during which the submandibular gland has the highest sensitivity to NE.

### Abundance of *Pigr* increases during the light phase and rhythmicity is lost following SCNX or *Clock* mutation

**Figure 5:**
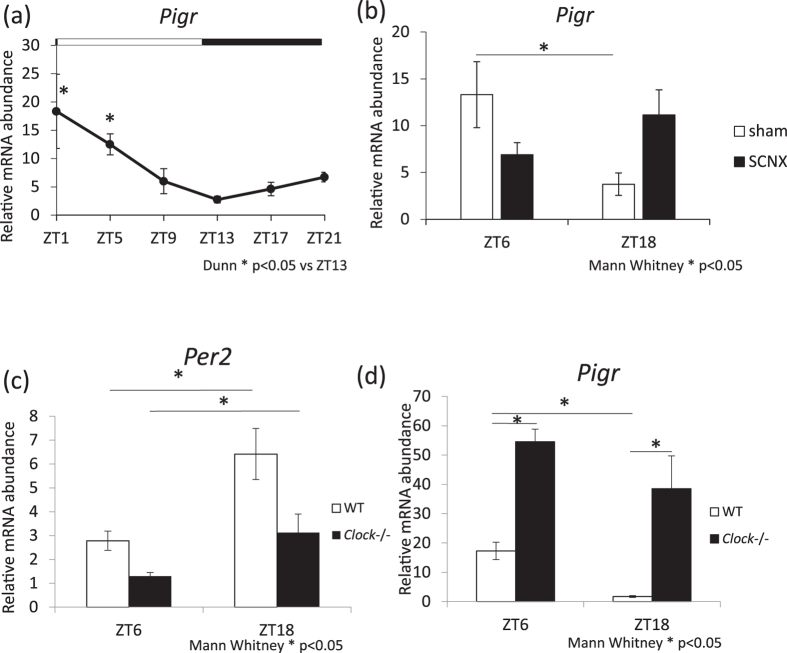
Circadian rhythm for *Pigr* gene abundance in submandibular glands in intact SCN-lesioned and *Clock* ^−/−^ mice. (**a**) Relative mRNA abundance rhythms of *Pigr* (n = 4) in the submandibular gland. (**b**) The relative mRNA abundance of *Pigr* in SCN-lesioned mice compared to that in sham-operated mice at ZT6 and ZT18 (n = 6–7). (**c**,**d**) The relative mRNA abundance of *Per2* (**c**) and *Pigr* (**d)** in *Clock* ^−/−^ mice compared to that in wild-type (WT) mice at ZT6 and ZT18 (n = 4 for each group). Values are shown as means ± SEM. (**a**) *p < 0.05, ZT1, ZT5 vs. ZT13 (Kruskal-Wallis test with Dunn post-hoc test), (**b**–**d**) *p < 0.05, ZT6 vs. ZT18 or WT vs. *Clock* ^−/−^ (Mann-Whitney test).

We investigated whether the rhythmicity of salivary IgA secretion is controlled by pIgR. The pIgR mediates transcytosis of IgA secretion; therefore, we investigated whether the abundance of the *Pigr* gene in the submandibular glands demonstrates circadian rhythms. We observed that *Pigr* abundance in the submandibular glands increased during the light phase, and peaked at ZT2.2 (Fig. 5a, Supplemental Table S2). To investigate the influence of the central circadian clock on *Pigr* abundance rhythms in the submandibular glands, we next performed a SCNX, which was compared with the sham group. In the sham group, there was a significant difference between ZT6 and ZT18, whereas no significant differences were observed among the SCNX groups (Fig. 5b). To determine whether the circadian rhythm of *Pigr* mRNA abundance is directly regulated by the *Clock* gene, *Pigr* mRNA abundance was examined using *Clock* ^−/−^ mice. The *Clock* mutation led to decreased *Per2* gene abundance in the submandibular gland (Fig. 5c). The levels of *Pigr* mRNA increased and their rhythm was lost upon *Clock* mutation (Fig. 5d). These results suggest that the abundance of *Pigr* fluctuates with the circadian rhythm, and this may be controlled by the SCN clock and *Clock* gene. Therefore, the rhythm of *Pigr* in the submandibular glands may influence salivary IgA secretion caused by sympathetic nerve activation.

### NE-induced salivary IgA secretion rhythm is maintained following adrenal gland lesion in mice

**Figure 6:**
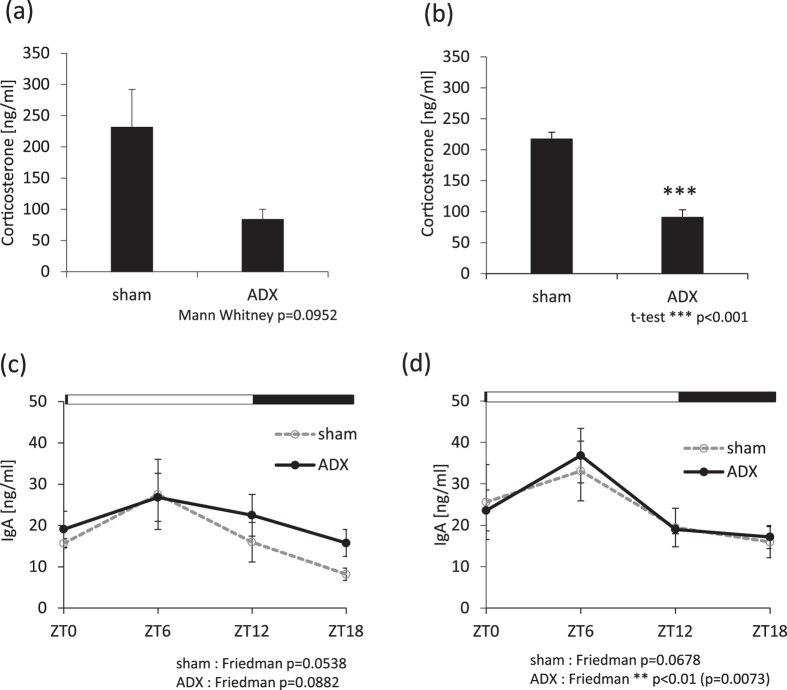
Effects of adrenal gland lesions on salivary IgA secretion. (**a**,**b**) Serum concentrations of corticosterone in the adrenal gland lesion (ADX) group compared to the sham-operated group for pilocarpine-only (**a**) and pilocarpine/NE-treated mice (**b**) (n = 3–6). (**c**,**d**) The rhythm of salivary IgA concentrations in the ADX group compared to that in the sham-operated mice for pilocarpine-only (**c**) and pilocarpine/NE-treated mice (**d**) (n = 3–6). (**a**) p = 0.0952 (Mann-Whitney test), (**b**) ***p < 0.001 (Student’s t-test), (**c**) sham, p = 0.0538 (Friedman test); ADX, p = 0.0882 (Friedman test), (**d**) sham, p = 0.0678 (Friedman test); ADX, **p < 0.01 (Friedman test).

It is well known that cortisol secretion in saliva is rhythmically controlled via the SCN clock, and secretion peaks during the early daytime hours^21^. External application of corticosterone or dexamethasone resets the phase activity of clock gene expression rhythms^22, 23^. Therefore, we investigated the contributions of corticosterone and adrenal gland activity to the NE-induced salivary IgA secretion rhythm. We prepared two sets of adrenal gland-lesion groups (pilocarpine and NE) to account for the possibility of recovery after the adrenal gland lesion. We lesioned the adrenal glands of mice, which resulted in decreased serum concentration of corticosterone (Fig. 6a,b). In sham-operated mice, groups treated with pilocarpine or pilocarpine and NE showed similar circadian IgA secretion (Fig. 6c,d, Supplemental Table S2), similar to that uninjured animals (Fig. 1a,d). In lesioned mice, pilocarpine alone or in combination with NE produced a pattern of salivary IgA circadian secretion similar to that observed in sham-operated mice (Fig. 6c,d, Supplemental Table S2). These data suggest that adrenal gland activity may not contribute to the NE-induced salivary IgA secretion rhythm.

## Discussion

In this study, we observed a clear circadian rhythm for salivary IgA concentration, total IgA content, and IgA per secreted protein in the saliva of NE- and pilocarpine-treated mice. These effects peaked during the light phase. A similar rhythm was seen in the pilocarpine-only group, but there were no significant peak values. Since salivary IgA concentrations increased following an additional NE administration, this suggested that NE signaling provides timing signals instructing salivary IgA secretion. We also concluded that this rhythm was not affected by saliva flow or total protein concentrations in saliva because these factors showed weak circadian rhythms. The rhythmicity of salivary IgA secretion was unaffected by 24-hour fasting, but the peak time was delayed. We previously showed that 24-hour fasting advanced the peripheral clocks evaluated by *in vivo* imaging of the bioluminescence rhythm in Per2::Luc mice^4^. Thus, rhythm formation of salivary IgA is not required for feeding or chewing.

There are two possible mechanisms for NE-induced salivary IgA secretion being the highest at ZT6 and the lowest at ZT18. One may be NE receptor sensitivity. In addition to our observations in Fig. 5, it has been reported that the submandibular gland shows circadian rhythms for clock gene expression^15, 16, 20^. We previously reported that the mRNA of adrenoceptors, including the α and β receptors, showed higher levels of expression during the light phase compared to that during the dark phase^19^, suggesting that external injection of NE has a strong effect during the light phase. The phase shifting effect on *Per2* gene expression rhythms in the salivary gland following daily NE injections was time-dependent. Our *in vitro* analyses demonstrated that NE directly affects clock function in the submandibular gland (Supplemental Fig. S5). Furthermore, significant advances in the phase after NE injections occurred with respect to the light phase, but not the dark phase. These data suggest that an inactive daytime period leads to a time window for external NE-induced salivary IgA secretion. The peak phases of IVIS data in ZT4 and ZT16 saline-treated groups demonstrated 2–3 hour differences between the two groups (Fig. 4). We previously reported that stress at an early inactive period caused phase advancement. Furthermore, stress at the early active period led to phase delay, suggesting that saline injection may be a stress stimulant, causing phase shifts of the submandibular gland clock.

Another mechanism involves the process of IgA secretion. It is known that pIgR mediates transcytosis of IgA^9^. Therefore, we investigated whether *Pigr* gene abundance levels in the submandibular glands show circadian rhythms. We found that *Pigr* expression in the submandibular glands increased during the light phase, suggesting that NE induced high salivary IgA secretion at ZT6. However, the IgA concentration in the submandibular gland showed weak circadian rhythms. These results suggest that the rhythm of *Pigr* abundance levels together with IgA concentration in the submandibular gland may influence the rhythmicity of salivary IgA secretion. These two mechanisms may act cooperatively to produce the observed daily rhythm of NE-induced salivary IgA secretion.

It is known that sympathetic nerve stimulation, such as through administration of isoproterenol, β-receptor agonists^14^, or NE decreases saliva flow. In addition, secretion of saliva is under sympathetic nervous control, and the proteins in saliva increase upon stimulation of the sympathetic nerve^24^. Therefore, it seems that the rhythmicity of salivary IgA secretion was not affected by the rhythms of saliva protein production. We observed that salivary IgA concentrations increased following treatment with an adrenoceptor agonist and decreased upon antagonist administration. In addition, both α and β receptor agonists increased salivary IgA secretion at both ZT6 and ZT18, and the peak in salivary IgA secretion during the light phase was still observed after treatment with both agonists. The stimulation of the sympathetic nervous system increased salivary IgA concentrations. However, it did not change the tendency for salivary IgA concentrations to be highest at ZT6 and lowest at ZT18. Pilocarpine-induced salivary IgA secretion showed a weak rhythmicity, and the highest levels were observed during the light phase. These results suggest that the sympathetic α and β receptor system mainly control the daily rhythm of salivary IgA secretion instead of the parasympathetic nerve system.

We found that the circadian rhythmicity of *Pigr* mRNA abundance in the submandibular glands was dampened in SCN-lesioned and *Clock* ^−/−^ mice. This strongly suggests that circadian signals from SCN and/or the circadian clock in the submandibular gland contribute to the NE-induced salivary IgA secretion rhythm. Vujovic *et al*. reported that the submandibular gland contains an autonomous clock that can persist in isolation from nervous input but is influenced by the sympathetic nervous system^17^. This report may support the requirement for sympathetic nerve activation for salivary clock function.

In human saliva, some substances such as cortisol, melatonin, and amylase show clear daily rhythms^21, 25, 26^. Therefore, we examined the possible interactions between NE-induced salivary IgA secretion and these substances. Similar to injections of NE^19, 27^, corticosterone caused phase-resetting in the peripheral clock of the salivary glands^22, 23^ in mice. Furthermore, stress decreased salivary IgA secretion in humans^28^. Interestingly, lesions of the adrenal gland in mice caused a pattern of pilocarpine- or pilocarpine-/NE-induced salivary IgA secretion similar to that in sham-operated mice. This suggests that NE-induced salivary IgA secretion is independent of adrenal gland function. We used the ICR strain of mice, which do not produce melatonin because of a mutation in an enzyme required for melatonin synthesis^29^. In this biological context, melatonin makes no contribution to salivary IgA secretion. To assess the role of amylase in the current experiments, we examined NE-induced salivary IgA secretion after 24 hours of fasting. The circadian pattern of salivary IgA secretion was very similar to that in the 6-hour fasting control group, suggesting a weak contribution of amylase to salivary IgA secretion.

It has been reported that a decrease in salivary IgA secretion may lead to URTI and periodontal disease^10^. We found that the amount of salivary IgA, which should increase during sleep, did not increase after disruption of the biological clock. These results suggest that salivary IgA does not increase when the biological clock is disturbed or when there is a higher risk of the aforementioned diseases. In addition, since the submandibular glands are controlled by the autonomic nervous system, salivary IgA secretion and saliva flow are modulated by sympathetic nervous stimulation compared to that by only parasympathetic nerve stimulation. Therefore, parasympathetic stimulation during the inactive sleep period may only weakly increase the basal secretion of IgA to protect from bacterial invasion. In our daily life, the sympathetic nervous system may become activated during inactive periods such as staying up late or under excessive stress, which leads to salivary IgA secretion. We speculate that activation of sympathetic nerves during sleep may protect from bacterial access to the epithelial surface through a significant secretion of IgA.

## Methods

### Animals

Animal care was conducted with permission from the Committee for Animal Experimentation at Waseda University (permission # 2016-A059), and in accordance with the Law (No. 105) and Notification (No. 6) of the Japanese Government.

Male ICR mice (7 weeks old) were purchased from Tokyo Laboratory Animals Science Co., Ltd. (Tokyo, Japan). For *in vivo* and *in vitro* monitoring of PER2::LUC bioluminescence, PER2::LUC mice (7 weeks old) were obtained, as previously described^19, 20, 22^. In some experiments, we used male ICR *Clock* mutant (*Clock* ^−/−^) mice and wild-type (WT) mice, and confirmed the genotypes by polymerase chain reaction^30^. The *Clock* mutation in mice was identified by an N-ethyl-N-nitrosourea mutagenesis screen of circadian periods^31^. Mice were housed at 22 ± 2 °C with a humidity of 60 ± 5% under a 12-hour light/dark cycle. The lights were turned on at 08:00 and turned off at 20:00. Mice were fed a normal commercial diet (Catalog #MF; Oriental Yeast Co., Ltd., Tokyo, Japan) and were given *ad libitum* access to water.

### Saliva collection

Mice were fasted for 6 hours before collection of saliva to exclude effects associated with feeding, and to ensure the consistency of the oral environment among experiments (except in Fig. 1g). In one experiment (Fig. 1g), mice were fasted for 24 hours to avoid any effects from feeding. Saliva was collected from the oral cavity using a micropipette after intraperitoneal injection of pilocarpine (1 mg/kg) (Wako Pure Chemical Industries Ltd., Osaka, Japan) to stimulate saliva secretion (366 ± 54 μL for 5 min) through parasympathetic nerve activation. NE and saline injection weakly stimulates saliva secretion (2.0 ± 1.0 μL for 5 min). Therefore, to examine the role of NE on IgA secretion, a mixed solution of pilocarpine (1 mg/kg) and NE (1 mg/kg) (Sigma-Aldrich, St. Louis, MO, USA.) (NE group) was used to stimulate the sympathetic nerves (232 ± 18 μL for 5 min), which was compared to the pilocarpine (1 mg/kg) injection alone group (control group). Drugs were dissolved in 1X phosphate buffered saline (PBS). PBS injection alone (0.2 ± 0.15 μL for 5 min) did not induce saliva secretion. Although we need at least 2.5 μL saliva for the IgA assay, a combination of PBS and NE injection did not yield enough volume of saliva. It has been reported that the aspiration method does not suppress the diurnal variation in salivary IgA secretion compared to the swab method in human experiments^32^. Therefore, we used pipettes to collect saliva. Saliva collection commenced 5 min after reagent injection. Saliva was placed in microcentrifuge tubes containing protease inhibitors and placed into an ice bath. Saliva samples were clarified by centrifugation at 16,000 × *g* for 10 min at 4 °C. Samples were stored at −80 °C until the analyses were performed.

### Drug treatment

We used prazosin hydrochloride (1 mg/kg) (Wako Pure Chemical Industries Ltd., Osaka, Japan) and propranolol hydrochloride (1 or 10 mg/kg) (TOKYO KASEI, Tokyo, Japan) as adrenoceptor antagonists. Phenylephrine (5 mg/kg) (Wako Pure Chemical Industries Ltd, Osaka, Japan) and isoproterenol (5 mg/kg) (Tocris Bioscience, Bristol, United Kingdom) were used as α receptor and β receptor agonists, respectively. Pilocarpine was mixed with 1X PBS and used as a control treatment.

### Tissue collection

Mice were euthanized and the submandibular glands were collected. Tissues were placed into protease inhibitor-containing microcentrifuge tubes, and incubated in an ice bath. Samples were homogenized using a micro-homogenizer and then centrifuged at 3,000 × *g* for 20 min at 4 °C. The supernatants were stored at −80 °C until the analyses were performed.

### IgA ELISA (Enzyme-Linked ImmunoSorbent Assay)

Salivary IgA levels were quantified using a Mouse IgA Ready-SET-Go! ELISA kit (eBioscience, Vienna, Austria), according to the manufacturer’s protocols.

### Protein content

The protein concentration of each saliva sample was quantified using a BCA Protein Assay Kit (Thermo Fisher Scientific, Waltham, MA, USA), using bovine serum albumin as a standard, following the manufacturer’s protocols.

### SCN lesion

To investigate the role of the SCN on salivary IgA secretion, we performed SCN lesions on mice. As described previously^20^, bilateral thermal lesions of the SCN were performed stereotaxically under anesthesia using midazolam/medetomidine hydrochloride/butorphanol tartrate. After surgery, locomotor activity displayed arrhythmicity in SCN-lesioned mice. We scarified mice to assess the histology of lesion sites by staining with cresyl violet, and to verify the extent of injury according to our previous paper^20^. A lesion electrode was inserted into in the cerebral cortex of mice in the sham group.

### Locomotor activity measurement

General locomotor activity was recorded with an infrared radiation sensor (F5B; Omron, Tokyo, Japan) as previously published^19, 20^. Double-plotted actograms of locomotor activity were recorded in 6-min epochs using ClockLab software (Actimetrics Ltd., Wilmette, IL, USA). Chi-squared periodogram analysis was used to examine the rhythmicity of behavioral data with the period (20–30 hr).

### Adrenal gland lesion

As described previously^19^, adrenal gland lesions were made using the dorsal approach under anesthesia with midazolam/medetomidine hydrochloride/butorphanol tartrate. Briefly, the skin on the back was shaved, and an incision of about 1 cm was made parallel to the spinal cord. The adrenal glands were removed via small openings made in the left and right muscle layers of the spinal cord. To maintain ion balance, mice with adrenal gland lesions were provided with 0.9% NaCl water *ad libitum*. A similar operation was performed on mice in the sham group, without removal of adrenal glands.

### Corticosterone ELISA

Serum corticosterone levels were quantified using the AssayMax Corticosterone ELISA Kit (Assay Pro, MO, USA) according to the manufacturer’s protocol.

### *In vivo* monitoring of PER2::LUC bioluminescence

*In vivo* monitoring of PER2::LUC bioluminescence was performed using an IVIS kinetics system (Caliper Life Sciences, Hopkinton, MA, USA, and Summit Pharmaceuticals International Corporation, Tokyo, Japan), as previously described^20^. Mice were anaesthetized with a mixture of isoflurane (Mylan Inc., Tokyo, Japan) and concentrated oxygen. Anesthetized mice were injected subcutaneously on the back near the neck with D-luciferin potassium salt (15 mg/kg; Promega, WI, USA). Among anesthetic drugs, isoflurane did not affect phase and free-running period of liver clock *ex vivo* monitoring of PER2::LUC mice^33^. Images were acquired using an *in vivo* imaging system (Perkin Elmer, Waltham, MA, USA) with a 1-min exposure time from the dorsal- and ventral-up positions 8 and 10 min after luciferin injection, respectively. Images were obtained six times a day at 4-hour intervals (ZT9, 13, 17, 21, 1, and 5). Mice were returned to their home cages between imaging sessions. Photon counts for each tissue were analyzed using the Living Image 3.2 software (Perkin Elmer). The average photons for the six time points for each day were designated as 100%, and the bioluminescence rhythm was expressed as a percentage of each set of six points for individual organs. The peak phase, amplitude, and rhythmicity of normalized data were determined using the single cosinor procedure program (Acro.exe version 3.5)^34^.

### Measurement of bioluminescence in *in vitro* cultures of submandibular glands from PER2::LUC mice

*In vitro* bioluminescence monitoring data were analyzed as previously described^34^. PER2::LUC mice were euthanized by cervical dislocation for the evaluation of bioluminescence rhythmicity in the submandibular glands. Submandibular gland sections on a membrane (Millicell cell culture inserts, Millipore, Billerica, MA, USA) were explanted into a 35 mm petri dish and cultured in 3.0 mL Dulbecco’s modified Eagle’s medium (DMEM, Invitrogen, Waltham, MA, USA) supplemented with NaHCO_3_ (2.7 mM), HEPES (10 mM), kanamycin (20 mg/L), insulin (5 μM/mL), putrescine (100 μM), human transferrin (100 μg/mL), progesterone (20 nM), sodium selenite (30 nM), and D-luciferin potassium salt (0.1 mM). Treatment with each reagent was at a specific time point between the first and second peak. Before the reagent was added, 3.0 mL of cultured medium was transferred to other dishes at 37 °C, and the membrane was transferred to each medium in turn (1 mL reagent medium for 30 min; 1 mL wash medium for 10 min; and 1 mL remaining medium for monitoring of bioluminescence). Bioluminescence was monitored for 1 min at 10-min intervals with a dish-type luminometer (LumiCycle, Actimetrics, IL, USA). The amplitude of the waveform was calculated using R software^35^ from the recorded data. The phase and period length were measured using Actimetrics software for LumiCycle with sin fitting as previously described^36^.

### Incubation of submandibular glands *in vitro*

Submandibular glands from ICR mice were incubated *in vitro* at 37 °C in modified Krebs-Ringer medium containing 0.2% glucose, and bubbled with a gas mixture of 95% O_2_ and 5% CO_2_. Krebs-Ringer medium was used in the treatment group containing 10 μM NE. Submandibular glands were incubated for 1 hour. After incubation, these tissues were collected and real-time RT-PCR was performed.

### Isolation of total RNA and real-time RT-PCR

Total RNA was extracted from tissues, using phenol. Aliquots of 50 ng of total RNA were reverse-transcribed and amplified using a One-Step SYBR reverse transcription PCR (RT-PCR) kit (TaKaRa Bio Inc, Shiga, Japan) in Piko Real (Thermo Fisher Scientific Inc, Kanagawa, Japan). Primer pairs were designed based on published data for the *Gapdh, Per1, Per2*, and *Pigr* genes. The relative levels of the target gene PCR products were normalized to that of *Gapdh*. The data were analyzed using the delta-delta Ct method. The primers for *Gapdh* were as follows: Gapdh-F, 5′-TGGTGAAGGTCGGTGTGAAC-3′; Gapdh-R, 5′-AATGAAGGGGTCGTTGATGG-3′. The primers for *Per1* were as follows: Per1-F, 5′-CAAGTGGCAATGAGTCCAACG-3′; Per1-R, 5′-CGAAGTTTGAGCTCCCGAAGTG-3′. The primers for *Per2* were as follows: Per2-F, 5′-TGTGTGCTTACACGGGTGTCCTA-3′; Per2-R, 5′-ACGTTTGGTTTGCGCATGAA-3′. The primers for *pIgR* were as follows: pIgR-F, 5′-AGTAACCGAGGCCTGTCCT-3′; pIgR-R, 5′-GTCACTCGGCAACTCAGGA-3′.

### Statistical analyses

Statistical analyses were performed using GraphPad Prism software, version 6.03 (GraphPad Software, San Diego, CA, USA). Equal variance and normal distribution tests were performed to select the appropriate statistical approach. Parametric analysis was conducted by one-way, one-way repeated or two-way ANOVA with a Tukey test and Student’s t-test. Non-parametric analysis was performed by a Kruskal-Wallis or Friedman test with Dunn and Mann-Whitney tests. The data are presented as means ± SEM. The value p < 0.05 was considered significant.

## Electronic supplementary material

Supplementary Information
