# Supplementary material for: Circadian clock-dependent increase in salivary IgA secretion modulated by sympathetic receptor activation in mice
Source: Sci Rep. 2017 Aug 18;7:8802. doi: 10.1038/s41598-017-09438-0 (PMC5562870; doi:10.1038/s41598-017-09438-0)
Supplement: Supplementary file 1 — Supplementary Information [file 41598_2017_9438_MOESM1_ESM.pdf]

Supplementary Information for

Misaki Wada, Kanami Orihara, Mayo Kamagata, Koki Hama, Hiroyuki Sasaki, Atsushi Haraguchi, Hiroki Miyakawa, Atsuhito Nakao, and Shigenobu Shibata

Circadian clock-dependent increase in salivary IgA secretion modulated by sympathetic receptor activation in mice

This PDF file includes
Figs. S1 to S5
Table S1 to S2

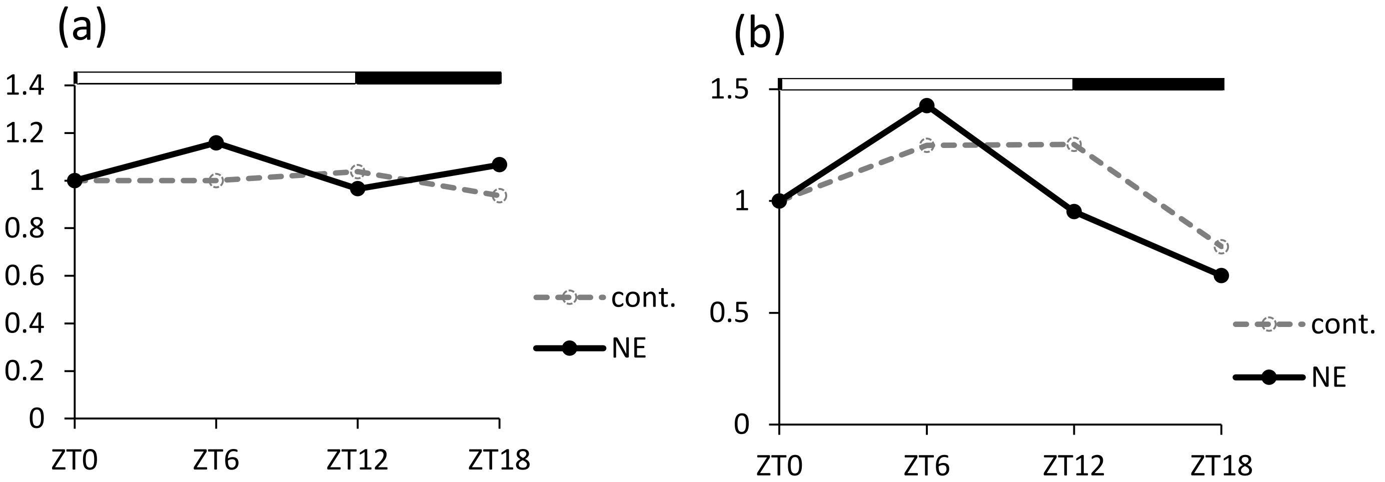

Supplementary Figure. S1 The circadian rhythm of the concentration ratio of IgA and protein in saliva.
(a) The circadian variation in the protein concentration ratio in saliva following administration of pilocarpine (control) versus a mixture of pilocarpine and NE. (b) Circadian variation in the salivary IgA concentration ratio in control versus NE groups (control, n=4; NE, n=9-10).

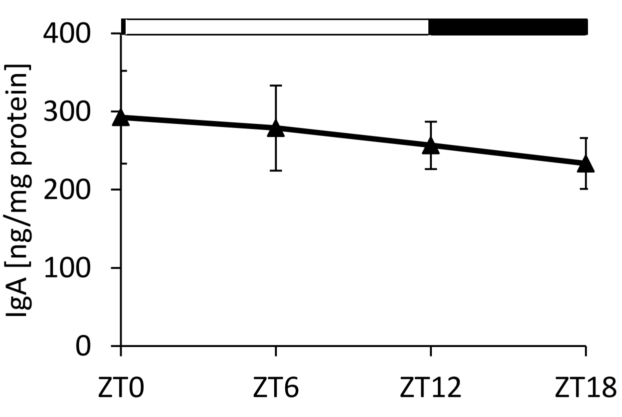

Supplementary Figure. S2 The circadian rhythm of submandibular gland IgA concentrations.
The circadian variation in IgA concentration in the submandibular glands (n=4-5 for each time point).

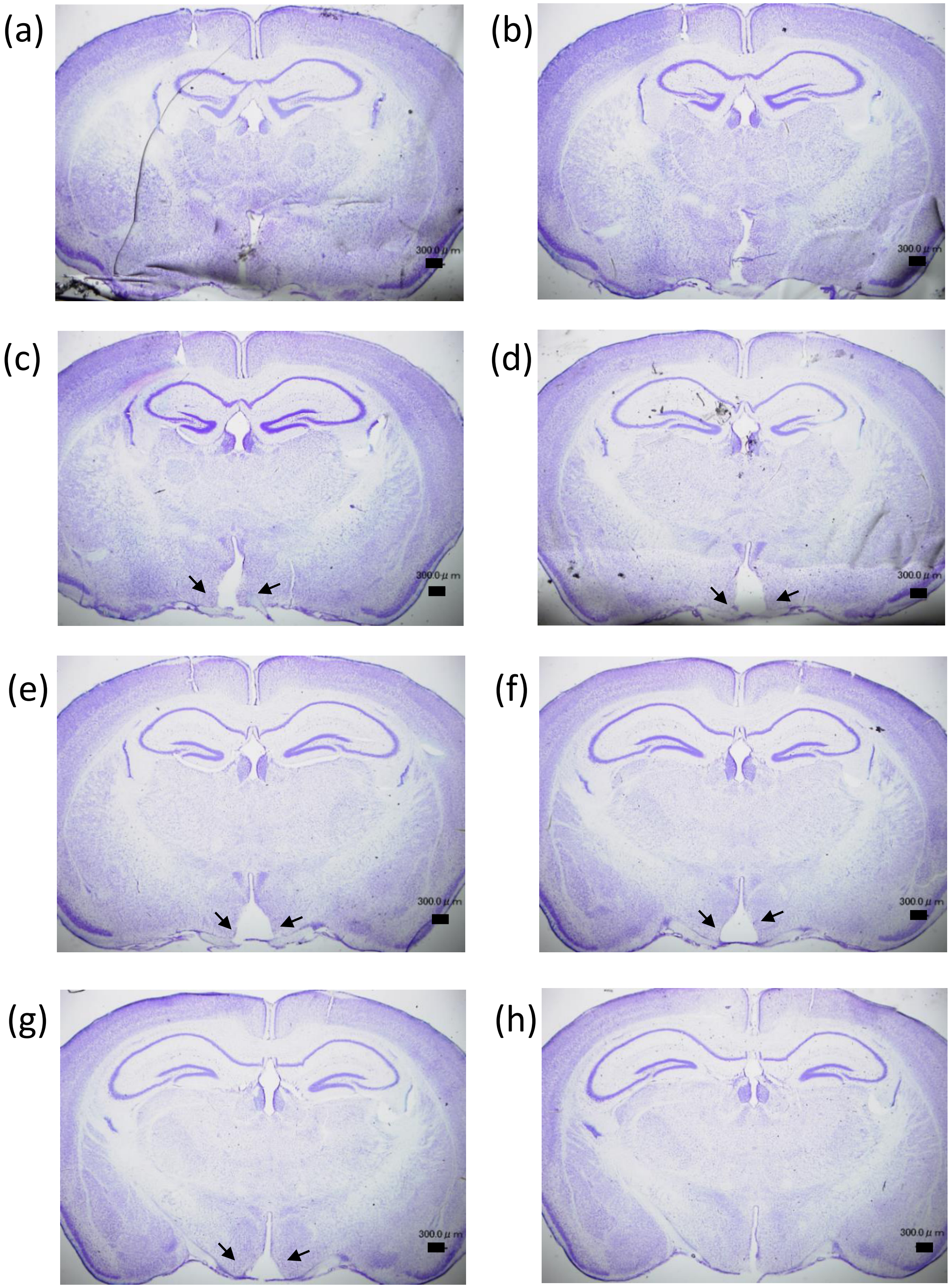

Supplementary Figure. S3 Representative example of histology of SCN lesion sites
(a)-(h) rostral to caudal section of SCN area. SCN was clearly lesioned in panels (c)to (g) indicated by arrows. Horizontal bar; 300 μM.

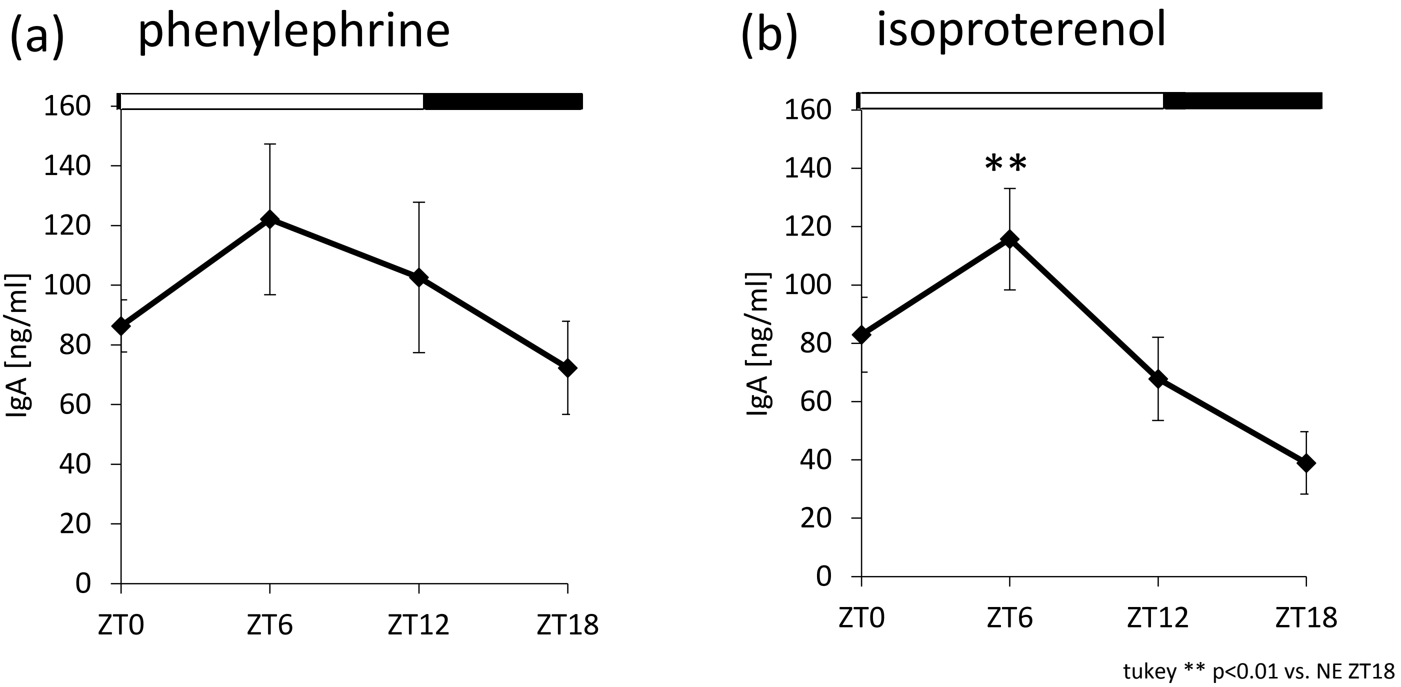

Supplementary Figure. S4 Phenylephrine- or isoproterenol- induced circadian rhythm of the concentration of IgA in saliva.

(a)(b) The circadian variation in concentration of IgA ratio in saliva following administration of mixture of pilocarpine (1mg/kg)+ phenylephrine (5mg/kg) or pilocarpine (1mg/kg) + isoproterenol (5mg/kg)(n=5 for each time point). ** $p < 0.01$, cont. group ZT6 vs. ZT18 (one-way ANOVA with Tukey post-hoc test).

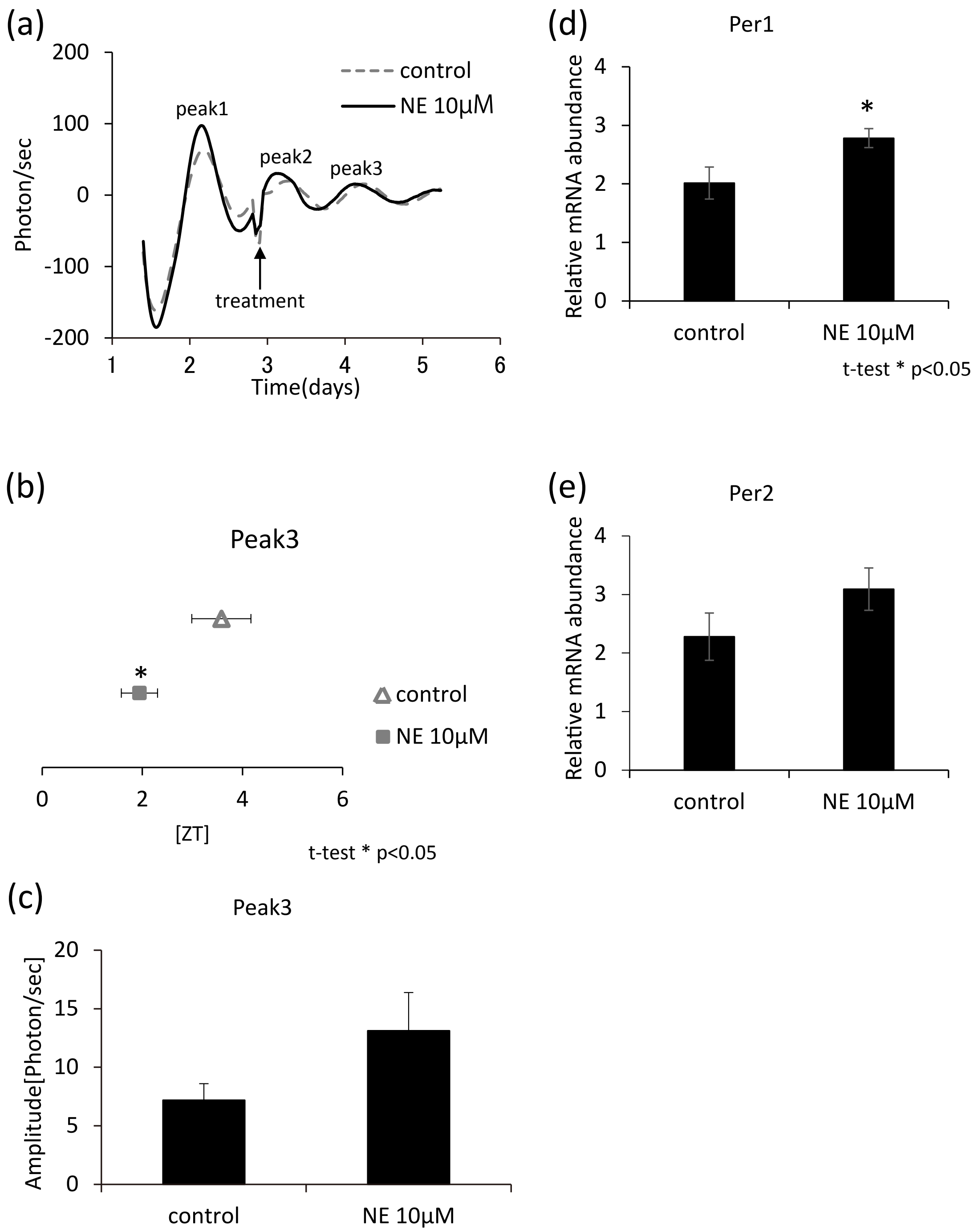

Supplementary Figure. S5 Effect of NE on phase and Per1 gene abundance in submandibular gland clock in vitro

(a) Wave forms of the bioluminescence rhythm in submandibular glands from PER2::LUC mice. NE or vehicle were applied at rising phase of rhythm after peak 1 (arrow) (b)(c) Average of phase shift and amplitude of PER2::LUC rhythms at peak 3 indicated in (a) (n=6). (d)(e) mRNA abundance of *Per1* and *Per2* in the submandibular gland one hour after NE application (n=6). * $p < 0.05$ (Student's t-test).

Supplementary T able S1. Summary of statistical analyses by two-way or one-way ANOVA

Figure No.	Two-way ANOVA					One-way ANOVA			
	Main effect A		Main effect B		Interaction	factor	group	test	P-value
	factor	P-value	factor	P-value	P-value				
1(a)			-			time	cont.	Kruskal-Wallis	p=0.397
							NE	One-way ANOVA	p=0.009
1(b)	time	p=0.885	treatment	p<0.001	p=0.846	time	cont.	One-way ANOVA	p=0.917
							NE	One-way ANOVA	p=0.635
1(c)			-			time	cont.	Kruskal-Wallis	p=0.227
							NE	One-way ANOVA	p=0.006
1(d)			-			time	cont.	Kruskal-Wallis	p=0.057
							NE	One-way ANOVA	p=0.009
1(e)	time	p=0.562	treatment	p<0.001	p=0.349	time	cont.	One-way ANOVA	p=0.835
							NE	One-way ANOVA	p=0.089
1(f)			-			time	cont.	Kruskal-Wallis	p=0.130
							NE	Kruskal-Wallis	p=0.026
1(g)			-			time	cont.	Kruskal-Wallis	p=0.130
							NE	One-way ANOVA	p=0.002
2(e)	time	p=0.123	treatment	p=0.085	p=0.585	time	sham	One-way ANOVA	p=0.128
							SCNX	One-way ANOVA	p=0.468
2(f)			-			time	sham	One-way ANOVA	p=0.002
							SCNX	Kruskal-Wallis	p=0.986
4(c)			-			time	control	Friedman	p<0.001
							NE 0.5mg/kg	Friedman	p<0.001
							NE 2mg/kg	Friedman	p<0.001
4(e)			-			time	control	Friedman	p<0.001
							NE 0.5mg/kg	Friedman	p<0.001
							NE 2mg/kg	One-way repeated ANOVA	p=0.003
5(a)			-			time	*Pigr*	Kruskal-Wallis	p=0.007
6(c)			-			time	sham	Friedman	p=0.054
							ADX	Friedman	p=0.088
6(d)			-			time	sham	Friedman	p=0.068
							ADX	Friedman	p=0.007
S1(a)	time	p=0.562	treatment	p<0.001	p=0.349	time	cont.	One-way ANOVA	p=0.835
							NE	One-way ANOVA	p=0.089
S1(b)			-			time	cont.	Kruskal-Wallis	p=0.397
							NE	One-way ANOVA	p=0.009
S2			-			time	IgA	One-way ANOVA	p=0.839
S4(a)			-			time	IgA	One-way ANOVA	p=0.357
S4(b)			-			time	IgA	One-way ANOVA	p=0.011

Hyphen in “Two -way ANOVA” column indicates that the data could not be analyzed by two-way ANOVA, because of non-normal distribution data, biased variation data or only one factor data.

Supplementary Table S2. Cosinor analysis of daily rhythms in each experiment.

Figure No.	Treatment	Acrophase(hr)	Amplitude	P-value
1(a)	cont.	7.8	6.713	p<0.02
	NE	6.0	19.85	p<0.005
1(b)	cont.	4.8	21.833	p<0.02
	NE	11.4	21.447	p<0.001
1(c)	cont.	6.0	3534.020	p<0.005
	NE	6.6	4687.647	p<0.001
1(d)	cont.	6.6	16.149	p<0.05
	NE	6.0	19.850	p<0.005
1(e)	cont.	7.8	133.772	p>0.05
	NE	4.8	376.916	p>0.05
1(f)	cont.	6.6	5.892	p<0.05
	NE	6.0	3.981	p<0.005
1(g)	cont.	4.8	5.357	p<0.02
	NE	4.2	32.689	p<0.05
2(e)	sham	5.4	2.976	p<0.01
	SCNX	2.4	1.91	p<0.01
2(f)	sham	8.4	30.343	p<0.005
	SCNX	13.8	9.935	p<0.05
4(c)	cont.	18.4	86.616	p<0.001
	NE 0.5mg/kg	17.1	106.707	p<0.01
	NE 2mg/kg	14.6	89.049	p<0.001
4(e)	cont.	20.9	75.259	p<0.001
	NE 0.5mg/kg	21.3	88.184	p<0.001
	NE 2mg/kg	20.9	71.765	p<0.001
5(a)	*Pigr*	2.2	7.802	p<0.01
6(c)	sham	6.0	9.668	p<0.005
	ADX	7.2	5.523	p<0.001
6(d)	sham	4.8	8.593	p<0.005
	ADX	5.4	9.828	p<0.05
S1(a)	cont.	7.8	0.051	p>0.05
	NE	4.8	0.096	p>0.05
S1(b)	cont.	7.8	0.229	p<0.02
	NE	6.0	0.381	p<0.005
S2	IgA	3.6	29.562	p<0.05
S4(a)	phenylephrine	7.2	24.908	p<0.001
S4(b)	isoproterenol	5.4	38.375	p<0.001

Acrophase was calculated by cosinor analysis.
